# Effectiveness of a Web-Based Tailored Intervention With Virtual Assistants Promoting the Acceptability of HPV Vaccination Among Mothers of Invited Girls: Randomized Controlled Trial

**DOI:** 10.2196/jmir.7449

**Published:** 2017-09-06

**Authors:** Mirjam Pot, Theo GWM Paulussen, Robert AC Ruiter, Iris Eekhout, Hester E de Melker, Maxine EA Spoelstra, Hilde M van Keulen

**Affiliations:** 1 Netherlands Organization for Applied Scientific Research (TNO) Child Health Leiden Netherlands; 2 Department of Work and Social Psychology Maastricht University Maastricht Netherlands; 3 VU University Medical Center Epidemiology & Biostatistics Amsterdam Netherlands; 4 National Institute for Public Health and the Environment (RIVM) Centre for Infectious Disease Control Bilthoven Netherlands; 5 Department of Psychology Leiden University Leiden Netherlands

**Keywords:** vaccination, health promotion, web-based intervention computer-tailoring, randomized controlled trial

## Abstract

**Background:**

In 2010, the human papillomavirus (HPV) vaccination was introduced in the Dutch National Immunization Program for 12-year-old girls, aiming to reduce the incidence of cervical cancer in women. HPV vaccination uptake turned out to be lower than expected: 61% versus 70%, respectively. Mothers were shown to play the most important role in the immunization decision about this vaccination. They had also expressed their need for interactive personal information about the HPV vaccination over and above the existing universal general information. To improve the effectiveness of the existing education about the HPV vaccination, we systematically developed a Web-based tailored intervention with virtual assistants providing mothers of girls to be invited with tailored feedback on their decision making about the HPV vaccination.

**Objective:**

The aim of this study was to evaluate the effectiveness of the Web-based tailored intervention for promoting HPV vaccination acceptance by means of a randomized controlled trial (RCT).

**Methods:**

Mothers were recruited via the Dutch vaccination register (Praeventis) (n=36,000) and three Web-based panels (n=2483). Those who gave informed consent (N=8062) were randomly assigned to the control (n=4067) or intervention condition (n=3995). HPV vaccination uptake, as registered by Praeventis once the HPV vaccination round was completed, was used as the primary outcome. Secondary outcomes were differential scores across conditions between baseline (before the provided access to the new tailored intervention) and follow-up (just before the first vaccination) regarding the mothers’ degree of informed decision making (IDM), decisional conflict, and critical determinants of HPV vaccination uptake among which are intention, attitude, risk perception, and outcome beliefs.

**Results:**

Intention-to-treat analysis (N=8062) showed a significant positive effect of the intervention on IDM, decisional conflict, and nearly all determinants of HPV vaccination uptake (*P*<.001). No effect was found on uptake (*P*=.60). This may be attributed to the overall high uptake rates in both conditions. Mothers evaluated the intervention as highly positive, including the website as well as the virtual assistants that were used to deliver the tailored feedback.

**Conclusions:**

This computer-tailored intervention has the potential to improve HPV vaccination acceptability and IDM and to decrease decisional conflict among mothers of invited girls. Implications for future research are discussed.

**Trial Registration:**

Trialregister.nl NTR4935; http://www.trialregister.nl/trialreg/admin/rctview.asp?TC=4935 (Archived by WebCite at http://www.webcitation.org/6srT7l9EM)

## Introduction

Worldwide, cervical cancer is the third most common cancer in women [1]. Persistent infection by the human papillomavirus (HPV) is the causative agent of cervical cancer [2]. In the Netherlands, yearly 750 new cases of cervical cancer are detected, of which 242 are with fatal consequences [3], despite the presence of a national cervical cancer screening program for women aged 30 to 60 years [4]. In 2008, the Health Council advised the Ministry to include the HPV vaccination for girls aged 12 years in the National Immunization Program (NIP) [5]. Initial implementation started with a catch-up campaign in 2009 for girls aged 13 to 16 years. From 2010, new cohorts of 12-year-old girls have been invited by the NIP to receive the HPV vaccination on a yearly basis. The municipal health services organize local sessions for group-based HPV vaccination, usually at large venues. This restricts the opportunity for interaction between the parent and girl with the professional. The vaccination itself is given by young health professionals (ie, medical doctors, nurses, or doctor’s assistants). The vaccination is voluntary and is offered free of charge. Complete vaccination includes 2 injections with a 6-month interval.

So far, HPV vaccination uptake in the Netherlands has remained lower than expected: 61% uptake in 2016 while 70% was targeted [6]. Research has indicated that mothers play the most important role in decision making about the girls’ immunization [7]. Currently, the regular invitation for the HPV vaccination comprises an introduction folder and a link to a website providing universal information about HPV and HPV vaccination. However, research has already indicated that mothers feel more in need for interactive personal information about the HPV vaccination, over and above this universal information [8]. To improve the existing educational strategy targeting HPV vaccination uptake, we developed a computer-tailored intervention with virtual assistants using the 6-step Intervention Mapping protocol for developing theory- and evidence-based health promotion interventions [9]. The intervention was aimed at Dutch mothers of girls to be invited for the HPV vaccination in 2015 (ie, girls born in 2002).

To date, only few tailored interventions to encourage HPV vaccination have been tested [10-13]. Three of these showed positive results. Hopfer [11] found that HPV vaccination uptake doubled among participants who were exposed to a culturally tailored video, compared with controls. Gerend and colleagues [12] found that, compared with general information, information tailored to the individual participant’s perceived barriers increased HPV vaccination intention. Grandahl and colleagues [13] found that an intervention delivered individually, that is face-to-face, by school nurses positively affected beliefs toward HPV prevention as well as vaccination uptake. However, to our knowledge, effective interventions promoting HPV vaccination that can reach large groups at relatively low costs (eg, Web-based tailored interventions) [14] do not exist yet.

Social cognitive determinants of the mothers’ decision making about their daughters’ HPV vaccination that appeared both relevant and changeable were selected as targets for developing the intervention [9]. These included HPV vaccination-related intention, attitude, outcome beliefs, risk perception, anticipated regret, subjective norms, habit, relative effectiveness of the HPV vaccination, and self-efficacy [7,8,15]. These determinants appeared to account for large proportions of variance in the mothers’ decision-making outcome (80-82%) [7]. Also, large proportions of the mothers do not actively acquire and process information about the pros and cons of the HPV vaccination and feel ambivalent about their decision [7,8]. This indicates that these decisions are based on rather unstable grounds, which makes them vulnerable for arguments challenging their initial attitudes and/or intention. Because informed decision making (IDM) is expected to make mothers less vulnerable for counterarguments, this was also chosen as a relevant intervention target. According to Marteau and colleagues [16], an informed decision is based in sufficient and relevant knowledge and in the congruence between the person’s values (ie, their attitude toward the HPV vaccination) and the behavioral outcome (ie, whether mothers had their daughters vaccinated against HPV). Consequently, knowledge was also targeted by the intervention. In addition, decisional conflict was selected as a target as this appears strongly related to IDM, with the possibility that decisional conflict may arise when feeling uninformed [17].

The aim of this study was to assess the effectiveness of the Web-based tailored intervention with virtual assistants on HPV vaccination uptake among the participants’ daughters (primary outcome). Secondary outcomes were the mothers’ degree of IDM, decisional conflict, and the social cognitive determinants of decision making about the daughters’ HPV vaccination uptake (eg, attitude, intention, and beliefs). When compared with the control condition, significantly positive effects were expected in the intervention condition with respect to HPV vaccination uptake, social cognitive determinants of the mothers’ decision making about the vaccination, levels of informed decision making, and levels of decisional conflict.

## Methods

### Participants

Mothers were randomly recruited from Praeventis, the Dutch National Immunization Register, and three Internet panels. The latter was to assure a high response rate [7]. This gave us the opportunity to assess differential effects of the intervention under (1) more controlled conditions (ie, panel sample) and (2) more naturalistic conditions as will be the case when the intervention has become part of the national implementation strategy (ie, Praeventis sample). This provided a basis for inferences concerning the intervention’s efficacy and effectiveness [18]. This is also why we did not reward the mothers from the Praeventis sample. The panel members received a small financial reimbursement for each survey that they completed (2-3 euros per survey). The amount of money received was panel specific. Extra financial reimbursement was provided to those in the intervention condition who completed all surveys (1-3 euros extra). In total, the participants in the control group could receive between 4 and 6 euros (completing baseline and follow-up), whereas participants in the experimental group could receive between 5 and 9 euros (completing baseline, intervention, and follow-up). Panel members were prestratified by region to ensure geographic diversity.

### Power Calculation

Power analyses indicated that 1200 mothers per sample (ie, Praeventis and panels) were needed at baseline (600 per arm) to detect a 10% difference in HPV vaccination uptake between the intervention and control group, and small effects on the continuous secondary outcomes (Cohen *d*=0.10-0.30), with a power of 0.80, a two-sided alpha of .05, and an expected dropout of 30% at the last survey. Given previous experiences [7,8], a response rate of 3% was expected in the Praeventis sample. A total of 36,000 mothers were initially invited to participate via Praeventis and 1200 mothers via the Internet panels.

### Design

This study, approved by the Ethical Committee of the VU Medical Center in Amsterdam, was conducted between January 2015 and March 2015. Effectiveness was evaluated by a 2-arm randomized controlled trial (RCT). Intervention effects on HPV vaccination uptake were assessed objectively using Praeventis. Because invited girls were given the opportunity to catch up on their missed HPV vaccinations, complete data on uptake were only available 18 months after baseline (ie, July 2016). The effects on secondary outcomes were examined by two Web-based surveys; at baseline, just before they had access to the experimental education, and at follow-up, just before they received the first HPV vaccination (time intervals around 2 months). Participants in the intervention condition were invited to visit the Web-based intervention between baseline and follow-up. Participants in both arms had access to the universal information about the HPV vaccination as part of the regular invitation for the HPV vaccination.

### Procedure

**Figure 1:**
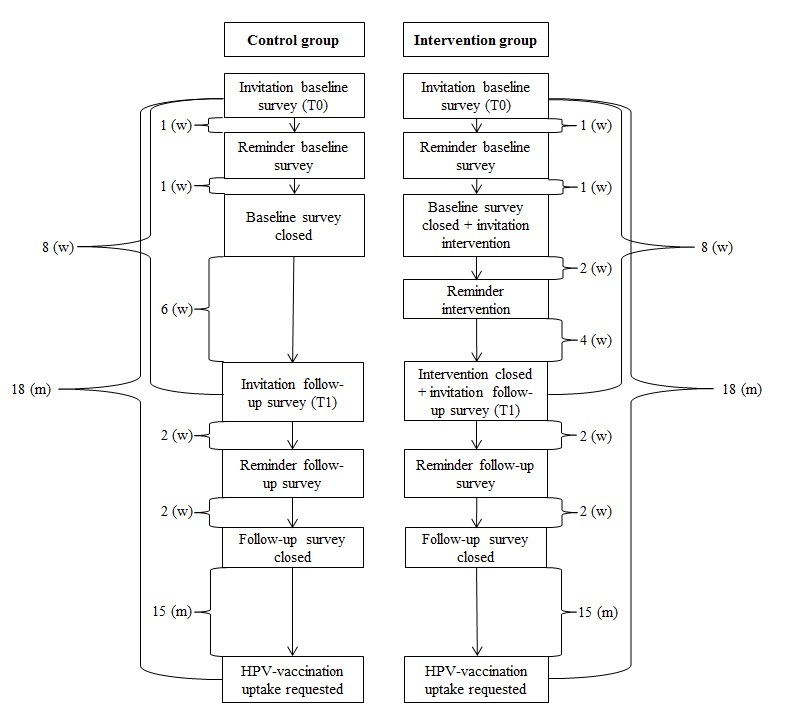
Timeline of data collection for the control and intervention group.

An invitation letter to participate in the study was sent in January 2015 by postal mail to the Praeventis sample and by email to the panel sample. This letter included information about the study, a link to a secured website, and a unique code for entrance to the baseline survey. The same code was used for entering the follow-up survey and for gaining access to the tailored intervention (only participants in the intervention condition). Securing the website and providing unique codes was done to reduce the risk of spillover effects. The mothers in the intervention condition were explicitly requested not to share the link with others. On the website, the participants were assured of their privacy, confidentiality, and security in handling their responses and were informed that they could withdraw from participation at any time. Participants were then asked to provide informed consent and to give us permission to derive their daughters’ HPV vaccination status from Praeventis. After having provided informed consent, participants were randomly assigned to either the control or intervention condition. A reminder was sent 1 week after the first invitation. One week after the reminder, participants in the intervention condition received an email inviting them to visit the Web-based tailored feedback. Two weeks after this invitation, a reminder was sent. The website could be visited until the invitation to complete the follow-up questionnaire, 8 weeks after the initial invitation for the baseline questionnaire. All participants were given 2 weeks to complete the follow-up survey; a reminder was sent after the first week. These timelines (see Figure 1) were chosen as these fit with the standard procedures for the HPV vaccination in the Netherlands.

### Intervention

**Figure 2:**
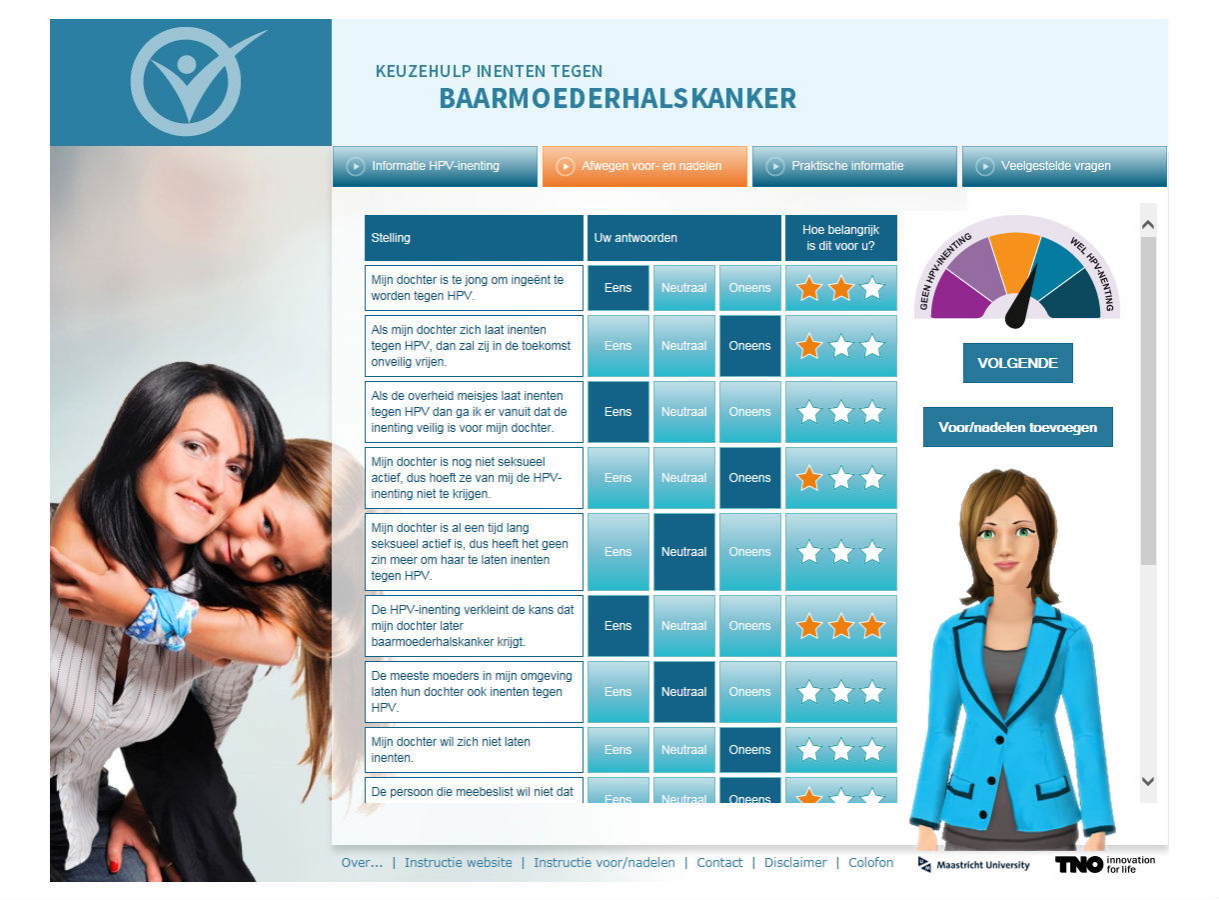
Screenshot of the decisional balance and the mother-like virtual assistant on the website.

The intervention consisted of a website providing mothers with tailored feedback from 2 virtual assistants, one being visualized in Figure 2. In Multimedia Appendix 1, a selection of screenshots of the website is presented. Computer-tailoring was the basic method for change and fitted the outcome of a previously conducted needs assessment indicating that the mothers preferred personalized feedback [8]. Tailoring is a health communication strategy in which messages are individualized to the person’s preferences and needs [19]. Meta-analyses have shown computer-tailored interventions to be more effective than universal interventions in achieving behavioral change outcomes (eg, [20,21]). Examples of change techniques that were used in addition to tailoring were consciousness raising (targeting risk perception) [22], belief selection (targeting beliefs) [23], active learning (targeting knowledge retention) [24], and motivational interviewing (targeting decisional conflict and attitude change) [25]. Before completing the concept intervention, we experimentally pretested three different intervention components [26,27] and conducted three focus groups for pretesting the prototype’s feasibility.

Also, innovative was the use of 2 virtual assistants for delivering the tailored feedback; a mother- and doctor-like assistant. They provided opportunities for two-way interactions and for creating a highly personal experience. The added value of using a virtual assistant over a text and/or picture-based website is that it improves information recall [28], transfer of learning [29], amount of learning [30], self-efficacy expectations, literacy, and behavioral change [31-33].

The website consisted of four menu options: (1) two-sided information about the HPV vaccination, (2) a decisional balance, (3) practical background information, and (4) frequently asked questions (see Multimedia Appendix 1). In the first menu, mothers were able to collect tailored information about the HPV vaccination such as information about the risk of contracting an HPV infection, which may cause cervical cancer, as well as the risks and effectiveness of the HPV vaccine. In the second menu, a decisional balance gave mothers the opportunity to weigh their perceived pros and cons to balance the mothers’ position toward vaccinating versus not vaccinating the daughter (see Figure 2). In the third menu, mothers received practical information such as how and where to get the HPV vaccination and how to talk to their daughter and/or partner about the HPV vaccination.

### Outcome Measures

#### Primary Outcome: HPV Vaccination Uptake

HPV vaccination uptake was derived from Praeventis, which was registered as having received no, 1, or 2 injections. We dichotomized HPV vaccination uptake into having received no HPV injection (0=not vaccinated) versus having received 1 or 2 HPV injections (1=vaccinated), as data showed that the determinants of HPV vaccination contrasted in these groups contrasted the most.

#### Secondary Outcomes: IDM and Decisional Conflict

According to Marteau and colleagues [16], a choice is considered to be informed when people have sufficient and relevant knowledge (knowledge) and when the person’s values (attitude) and behavior match. As such, IDM is usually expressed dichotomously (eg, see [34]). However, as we think the selection of the cut-off points is somewhat arbitrary, yet critical for the outcome [35], we also constructed a continuous measure for IDM. Post hoc analyses showed the correlation to be high between the two (Spearman ρ [rho]=.78). Both scores were derived from the Multi-dimensional Measure of Informed Choice [16,34,36].

By constructing the dichotomous IDM, the mother was classified as an informed decision maker, if (1) she had sufficient knowledge and (2) her attitude was consistent with the behavior (HPV vaccination uptake). Knowledge was considered to be sufficient when it was higher than the baseline mean score. A decision was considered to be consistent when the mother’s attitude was positive (higher than 4 on a 7-point scale) and the daughter was vaccinated, or when her attitude was negative (lower than 4 on a 7-point scale) and her daughter was not vaccinated. Any other combination was categorized as an uninformed decision.

By constructing the continuous measure of IDM, we first recoded the attitude scale into a scale ranging from −3 (extremely negative) to 3 (extremely positive). Consistency (ranging from −3 to 3) resulted from multiplying this attitude score with uptake (−1=no injection vs 1=1 or 2 injections). Then, the resulting consistency score was recoded from 0 (least consistent) to 6 (most consistent). Next, knowledge (−8 to 8, see Table 1) was recoded into a scale ranging from 0 (least knowledgeable) to 8 (most knowledgeable); the original scores below 0 were all recoded to 0. Finally, the continuous scale for IDM was computed by multiplying knowledge (range 0-8) with consistency (range 0-6), resulting in a scale ranging from 0 (not/least informed) to 48 (most informed).

Decisional conflict was measured using the “Uncertainty” subscale of the Decisional Conflict Scales [37], which included three 7-point scaled items (eg, “as regards to the HPV-vaccination, I felt sure about my choice) (1=completely disagree to 7=completely agree). Internal consistency was high (Cronbach alpha=.94).

### Determinants of HPV Vaccination Uptake

Composite scores were computed for determinants of HPV vaccination uptake in case the scaled items showed internal consistency (Cronbach alpha >.60 or Pearson *r*>.64). See Table 1 for an overview of the primary and secondary outcome measures. All scores on scaled items were averaged into a scale because they showed sufficient internal consistency (Cronbach alpha ≥.78/Pearson *r* ≥.64); Cronbach alpha was used for scales consisting of more than 2 items, whereas Pearson *r* was used for scales consisting of 2 items; items with an (R) were reverse coded.

**Table 1:** Overview of primary and secondary outcome measures.

Measures and items					Score/scale (minimum to maximum value)	Cronbach alpha (α) or Pearson *r* (*r*)^2^	References
**Primary outcome**							
	**HPV** ^a^ **vaccination uptake**				0=not vaccinated, 1=vaccinated	N/A^b^	
		Uptake of the HPV vaccination is obtained through data from Praeventis.					
**Secondary outcomes**							
	**IDM** ^c^ ** outcome (dichotomous)**				0=not informed, 1=informed	N/A	[16], [34], [36]
		An informed decision has been made when:					
			The knowledge score was higher or equal to the mean of knowledge at baseline, the attitude score was higher than 4 (positive) and the HPV vaccination has been received.				
			The knowledge score was higher or equal to the mean of knowledge at baseline, the attitude score was lower than 4 (negative), and the HPV vaccination has not been received.				
			Any other combination was categorized as an uninformed decision.				
	**IDM outcome (continuous)**				0=least informed decision to 48=most informed decision	N/A	[16], [34], [38]
		Attitude was recoded from 0-7 to −3 (negative) to 3 (positive attitude) and HPV uptake was recoded from 0 or 1 to −1 (no injection) or 1 (1 or 2 injections).					
		Level of consistency was measured by multiplying the scores for attitude by those for HPV uptake (−3=low consistency; 3=high consistency). Consistency was then recoded into 0 (low) to 6 (high). Both consistency and sufficient knowledge were considered prerequisite for an informed decision.					
		Knowledge scores (−8=low; 8=high) lower or equal to 0 were considered insufficient (0=no insufficient knowledge; 8=high knowledge).					
		The level of IDM outcome was determined by multiplying the scores for knowledge with those for consistency.					
	**Decisional conflict about the HPV vaccination**				1=high to 7=low decisional conflict	.94 (α)	[17]
		As regards the HPV vaccination:					
			I felt sure about my choice				
			The decision was relatively easy to make				
			I was clear about the best choice for my daughter				
	**HPV vaccination intention**				1=low intention to vaccinate to 7=high intention to vaccinate	.92 (*r*)	[7,8]
		Are you planning on getting your daughter vaccinated against HPV?					
		How big is the chance that you will get your daughter vaccinated?					
	**Attitude toward the HPV vaccination**				1=negative to 7=positive attitude	.98 (α)	[38]
		Vaccinating my daughter against HPV is...					
			very undesirable to very desirable				
			very bad to very good				
			very negative to very positive				
			very unimportant to very important				
	**Risk perception (having received no HPV vaccination)**				1=low to 7=high risk perception	N/A	[38,39]
		Imagine that your daughter was not vaccinated against HPV.					
			The chance that my daughter will get cervical cancer is...				
	**Risk perception (having received the HPV vaccination)**				1=low to 7=high risk perception	N/A	[38,39]
		Imagine that your daughter was vaccinated against HPV.					
			The chance that my daughter will get cervical cancer is...				
	**Anticipated regret about rejecting the HPV vaccination**				1=low to 7=high anticipated regret	N/A	[7,8]
		Imagine your daughter has not received the HPV vaccination and she gets cervical cancer in the future.								
			How much would you regret your decision to let her receive no vaccination?	
	**Beliefs about the HPV vaccination**				1=negative to 7=positive beliefs about the HPV vaccination	.85 (α)	[39,40]
		If the government offers the vaccination, I assume it will be safe.					
		Our government shows responsibility for the health of the Dutch population by introducing the HPV vaccination.					
		The HPV vaccination was only introduced because the pharmaceutical industry will earn a lot of money from it (R).					
		There is too little known about whether the HPV vaccination effectively protects against cervical cancer (R).					
		There is too little known about the detrimental side effects of the HPV vaccination (R).					
		My daughter is too young to receive the HPV vaccination (R).					
		My daughter does not need the vaccination because she is not yet sexually active (R).					
	**Subjective norms toward the HPV vaccination** ^d^				−20=negative to 20=positive	.64 (*r*)	[38]
		Normative beliefs:					
			Regarding the HPV vaccination of your daughter, what is your expectation on the opinion of...				
				Social referents: partner^e^, daughter			
		Motivation to comply:					
			How motivated are you to comply with the opinion of...?				
	**Habit strength toward the HPV vaccination**				1=weak to 7=strong habit strength	.78 (*r*)	[41]
		Letting my daughter receive the HPV vaccination is something I do:					
			automatically				
			without thinking				
	**Self-efficacy expectations toward the HPV vaccination**				1=low self-efficacy to 7=high self-efficacy	.82 (α)	
		To what extent would you succeed in dealing with the following statements?					
			Guiding my daughter in the decision regarding the HPV vaccination				
			Having a good talk with my daughter about the HPV vaccination				
			Having a good talk with my partner^e^ about the HPV vaccination				
			Motivating my daughter to have herself vaccinated				
			Getting the actual HPV vaccination/2 injections with my daughter				
	**Knowledge about the HPV vaccination** ^f^				−8=incorrect, 8=correct	N/A	[7,8]
		Are the following statements true or false?					
			HPV is sexually transmittable.				
			Condoms fully protect against HPV.				
			My daughter is obliged to get the HPV vaccination when she is invited.				
			You will always notice when you are infected by HPV.				
			Only women can get infected by HPV.				
			Women who received the HPV vaccination are still advised to participate in the cervical cancer screening in the Netherlands.				
			The HPV vaccination fully protects against cervical cancer.				
			My daughter does not need to get the HPV vaccination if she is already sexually active.				
	**Relative effectiveness of the HPV vaccination** ^g^				−9=HPV vaccination least effective to 9=HPV vaccination most effective	N/A	[7,8]
		How would you rate the effectiveness of the following methods of preventing cervical cancer:								
			having safe sex				
			having sex with only 1 person in a lifetime				
			participating in the cervical cancer screening				
			having a healthy lifestyle (eg, not smoking)				
			the HPV vaccination				
			Participants rated the effectiveness of each method				

^a^HPV: human papillomavirus.

^b^N/A: not applicable.

^c^IDM: informed decision making.

^d^The subjective norms score was first computed by multiplying normative beliefs and motivation to comply for each social referent, and then by summing up the multiplications of the social referents.

^e^Only applicable if the mother indicated that she had a partner.

^f^Knowledge is not a scale because the answer on 1 item does not predict the answer on other items; the items were summed up to present a sum score of knowledge.

^g^The difference between the rated effectiveness of the HPV vaccination and the most effective alternative represented the relative effectiveness score (−9=HPV vaccination least effective to 9=HPV vaccination most effective).

### Sociodemographics

Sociodemographics were modeled as background variables (ie, age, educational level, country of birth, and religion). Level of education referred to the highest level the mother had completed. Educational level was classified into low (less than secondary or vocational education), intermediate (secondary through preuniversity education) or high (professional or university education) [7,8]. Country of birth was dichotomized into “Netherlands” versus “other,” as in our sample only 562 (6.97%) of 8062 (100%) mothers appeared to be born in a country other than the Netherlands. Religion was measured by asking the mothers about their religious convictions (Protestant, Roman Catholic, Muslim, Jewish, Buddhist, Hindu, other, or no religion). This was later classified as “Protestant” versus “not Protestant” as Protestants most refrain from vaccination compared with other religious or nonreligious groups in the Netherlands [7,8].

### Subjective Program Evaluation and Objective Program Use

Subjective program evaluation was assessed at follow-up for mothers in the intervention condition by asking them to evaluate the website and the virtual assistants on a 10-point scale; the higher the score, the more positive the evaluation. Objective program use was evaluated by computer logs assessing the number of visits and amount of time logged in per session. If participants logged in more than once, these were summed.

### Statistical Analyses

Descriptive statistics were used to describe the baseline sample. For analyzing the effects of the intervention, we used intention-to-treat (ITT) instead of complete case analysis. By using ITT, power increases while the risk of bias possibly caused by selective dropout decreases [42]. To deal with missing data, we applied multiple imputation by chained equations [42,43]. There were 15 imputed datasets generated using the predictive mean matching algorithm in Statistical Package for the Social Sciences (SPSS, IBM Corp) . The results from the imputed datasets were pooled together using Rubin’s rules [37]. Convergence of the imputations was checked by inspecting the iteration plots.

Intervention effects were examined by logistic and linear regression analyses (for dichotomous and continuous variables, respectively) by using the outcome at the follow-up as the criterion and the outcome-score at baseline and condition as the independent variables [44]. In view of multiple testing, an effect was considered significant when *P*<.003 (Bonferroni corrected alpha=.05/15 factors). The odds ratio was used as an indicator for effect size (Bonferroni corrected alpha=.05/14 factors). Effect sizes for the linear regressions were calculated in R (R Development Core Team) [45] using Cohen ƒ^2^ statistic, (*R*^2^*_AB_* – *R*^2^*_A_*)/(1 – *R*^2^*_AB_*), in which *B* is the variable of interest (ie, condition), *A* is the set of all other variables (ie, the outcome at baseline), *R*^2^*_AB_* is the proportion of variance accounting for *A* and *B* together, and *R*^2^*_A_* is the proportion of variance accounted for by *A*. These were interpreted as 0.02=small, 0.15=medium, 0.35=large [46]. Complete case analyses were performed as a sensitivity check for substantial differences with the results based on ITT.

Furthermore, we performed exploratory moderation analysis (Bonferroni corrected alpha=.05/15) to examine differences in effects regarding sociodemographics (ie, age, country of birth, education level, and religion) and sample (ie, Praeventis vs panels) by including a two-way interaction term (eg, condition × sample) in each of the aforementioned regression analyses [47]. In addition, we explored whether intention (at baseline) moderated the effects found in the primary and secondary outcomes. For this, intention was divided into three subgroups: (1) mothers with a negative intention (scores below half a standard deviation (SD) below the centered mean score of intention at baseline), (2) mothers who were hesitating (scores between half an SD below and above the centered mean of intention at baseline), and (3) mothers with a positive intention (scores more than half an SD above the centered mean score). Finally, subjective evaluations and objective use of the program were assessed by using descriptive analysis. IBM statistical package SPSS version 23 was used for analyzing the data [44].

## Results

### Response Rates and Attrition

**Figure 3:**
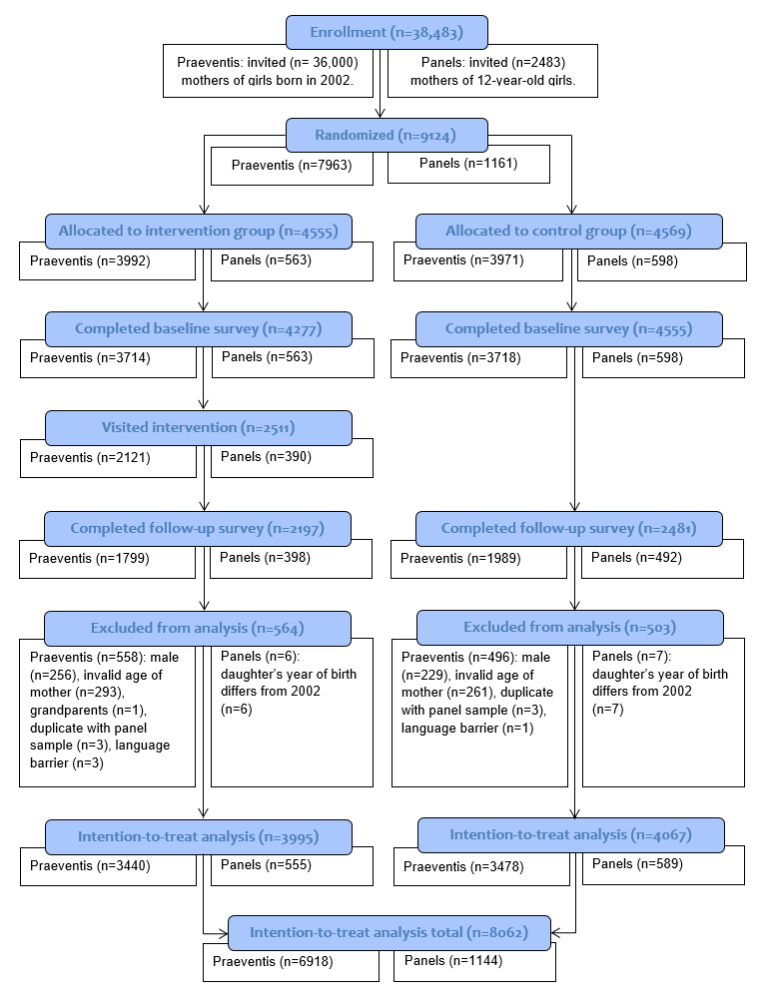
Flow diagram of the recruitment and response of study participants. Notes: (1) Participants could be excluded based on multiple criteria (eg, a male with an invalid age). Therefore, the total number of Praeventis participants excluded differed from the sum of separate criteria for exclusion. (2) In order to assess the intervention’s effectiveness (Praeventis sample) versus efficacy (panel sample), the recruitment and response is displayed per sample within each condition.

We invited 36,000 participants via Praeventis and 2483 via the panels. A flow diagram of the recruitment and response is shown in Figure 3. From the 9124 participants who were initially randomized at T0, 8593 (94.18%; 4277 in the intervention group and 4316 participants in the control group) completed the baseline questionnaire, whereas 4678 (51.27%; 2197 in the intervention group and 2481 participants in the control group) completed the follow-up questionnaire 8 weeks later (T1). Dropout analysis showed significantly more dropout in the Praeventis sample. There was also selective nonresponse with regard to condition (ie, more dropout in the intervention condition), sociodemographics (ie, more dropout in those not born in the Netherlands, and in those low in education), HPV vaccination uptake (more dropout in mothers having a daughter not being vaccinated), and secondary outcomes (ie, more dropout in mothers with low levels of IDM, risk perception, and self-efficacy and in mothers with high attitude scores). In total, 1067 participants were excluded (564 in the intervention group and 503 participants in the control group), as they did not meet the inclusion criteria (ie, being a mother of a daughter born in 2002 and aged 24-62 years) or were found to be duplicates across the two samples. The final sample for ITT analysis consisted of 8062 mothers: 3995 mothers in the intervention condition versus 4067 in the control condition.

### Sample Description

See Table 2 for the sample description. As there were no data available on sociodemographics of the population from which the sample was derived (ie, Dutch mothers of girls aged 12 years in the Praeventis database), we were unable to assess the representativeness of the study sample. The mean age of mothers was 43.64 years (SD 4.25). On average, mothers had a positive intention toward the HPV vaccination of their daughter at baseline (mean 5.35 [SD 1.69]). Compared with the national HPV vaccination uptake, uptake was higher in the study sample (n=59,866; 60.98% vs n=5880; 72.93%, respectively).

**Table 2:** Sample description (N=8062). In case of missing values, the number of missing values (N_missing_) was presented. By reporting 2 decimal points for the percentages, summing the percentages for each category up may differ from 100%.

Variables		Intervention (n_total_=3995), n (%)	Control (n_total_=4067), n (%)	Total (N_total_=8062), n (%)
Age in years, n (%)		43.70 (4.27)	43.58 (4.22)	43.64 (4.25)
**Country of Birth, n (%)**		N_missing_=4 (0.10)	N_missing_=4 (0.10)	N_missing_=8(0.10)
	The Netherlands	3715 (92.99)	3777 (92.87)	7492 (92.93)
	Other	276 (6.91)	286 (7.03)	562 (6.97)
**Religion, n (%)**		N_missing_=7 (0.18)	N_missing_=6 (0.15)	N_missing_=13 (0.16)
	Protestant	753 (18.85)	737 (18.12)	1490 (18.48)
	Not protestant	3235 (80.98)	3324 (81.73)	6559 (81.36)
**Educational level, n (%)**		N_missing_=4 (0.10)	N_missing_=3 (0.07)	N_missing_=7 (0.09)
	Low	588 (14.72)	540 (12.28)	1128 (13.99)
	Middle	1736 (43.45)	1735 (42.66)	3471 (43.05)
	High	1660 (41.55)	1786 (43.91)	3446 (42.74)
HPV^a^ vaccination uptake, n (%)		2923 (73.17)	2957 (72.71)	5880 (72.93)

^a^HPV: human papillomavirus.

### Intervention Effects on Primary and Secondary Outcomes

In Table 3, an overview of the intervention effects on the primary and secondary outcomes is given. A higher mean score means a higher X (eg, more positive attitude) except for decisional conflict; here, a higher mean score means less decisional conflict If an odds ratio (OR) is higher than one, this means that the higher the score on a factor, the higher the outcome of IDM or the higher the chance of the daughter being vaccinated. If an OR is less than one, this means that the higher the score on a factor, the lower outcome of IDM or the lower the chance of the daughter being vaccinated. ITT analyses showed that there was no effect of the intervention on HPV vaccination uptake (odds ratio, OR=1.03, *P*=.60). The intervention had a significant positive effect on all secondary outcomes (*P*<.001), except for risk perception when not vaccinated, anticipated regret, and self-efficacy (*P*=.01; *P*=.01; *P*=.03, respectively). Compared with the control group, at follow-up, mothers in the intervention group were more informed (dichotomous measure: OR=1.28, *P*<.001; continuous measure: beta=1.72, *P*<.003), experienced less decisional conflict (beta=.21, *P*<.003), were more intended to vaccinate their daughter (beta=.18, *P*<.004), had a more positive attitude toward vaccinating their daughter (beta=0.15, *P*<.004), had more positive beliefs (eg, beliefs about the safety and effectiveness of the HPV vaccination; beta=.12, *P*<.003), had a lower risk perception when they imagined that their daughter was vaccinated (beta=−.11, *P*<.003), perceived more positive subjective norms (beta=.82, *P*<.003), reported a higher relative effectiveness (beta=.46, *P*<.003), and had more knowledge (beta=.35, *P*<.003). Effect sizes were small (see Table 3). Results from complete case analyses were similar, except for an additional effect of the intervention on anticipated regret and self-efficacy.

**Table 3:** Effects of the intervention on the outcome measures according to intention-to-treat analyses (N=8062).

Outcome				Control (N=4067)				Intervention (N=3995)			Beta (standard error)		Cohen ƒ^2^ or OR	
				Pretest		Posttest		Pretest		Posttest				
Primary outcome														
	**HPV vaccination uptake** ^a^													
		Has received no HPV injection (reference), n (%)			1106 (27.19)				1066 (26.67)					
		Has received one or two HPV injections, n (%)			2961 (72.81)				2929 (73.32)		.03 (.05)^b^		1.03	
Secondary outcomes														
	**IDM: dichotomous**													
		Not informed (reference), n (%)	2689 (66.12)		1924 (47.31)		2689 (67.31)		1699 (42.53)					
		Informed, n (%)	1376 (33.83)		2143 (52.69)		1306 (32.69)		2296 (57.47)		.25 (.06)^c^		1.28	
	IDM: continuous (0-48), mean (SD)			18.95 (11.45)		24.28 (11.82)		18.69 (11.21)		25.85 (12.30)		1.72 (.27)^c^		0.007
	Decisional conflict (1-7), mean (SD)			4.33 (1.74)		5.17 (1.45)		4.33 (1.75)		5.38 (1.36)		.21 (.04)^c^		0.008
	Intention (1-7), mean (SD)			5.35 (1.70)		5.42 (1.97)		5.35 (1.69)		5.59 (1.87)		.18 (.03)^c^		0.006
	Attitude (1-7), mean (SD)			5.19 (1.46)		5.22 (1.57)		5.18 (1.45)		5.37 (1.51)		.15 (.03)^c^		0.006
	Beliefs (1-7), mean (SD)			4.21 (.72)		4.37 (.80)		4.19 (.73)		4.47 (.81)		.12 (.02)^c^		0.010
	Risk perception not vaccinated (1-7), mean (SD)			3.73 (.98)		3.70 (1.05)		3.74 (0.98)		3.77 (1.08)		.06 (.02)^d^		0.001
	Risk perception vaccinated (1-7), mean (SD)			2.76 (1.06)		2.74 (1.08)		2.77 (1.07)		2.64 (1.10)		−.11 (.03)^c^		0.004
	Anticipated regret (1-5), mean (SD)			3.68 (1.27)		3.50 (1.33)		3.71 (1.25)		3.59 (1.31)		.07 (.03)^d^		0.001
	Subjective norm (−20 to 20), mean (SD)			5.92 (7.90)		6.46 (9.46)		5.88 (7.81)		7.25 (9.20)		.82 (.20)^c^		0.004
	Habit (1-7), mean (SD)			4.26 (1.79)		4.36 (1.82)		4.28 (1.78)		4.51 (1.83)		.14 (.04)^c^		0.004
	Relative effectiveness (1-10), mean (SD)			−2.01 (2.24)		−1.84 (2.36)		−1.97 (2.22)		−1.35 (2.27)		.46 (.07)^c^		0.015
	Self-efficacy (1-7), mean (SD)			6.24 (.76)		6.24 (.78)		6.27 (.73)		6.29 (.75)		.04 (.02)^e^		0.001
	Knowledge (−8 to 8), mean (SD)			4.42 (2.16)		5.41 (2.09)		4.40 (2.14)		5.75 (2.09)		.35 (.05)^c^		0.009

^a^Human papillomavirus (HPV) vaccination uptake was not assessed at baseline.

^b^*P*=.60.

^c^*P* ≤.001, thus significant (*P*<.003; Bonferroni: 0.05/14 factors).

^d^*P*=.01.

^e^*P*=.03.

### Moderation of Intervention Effects

Regarding sociodemographics, no significant interaction effects on any of the outcome measures were found for country of birth (*P* ≥.08) or religion (*P* ≥.08). For educational level, we found an interaction effect with condition on relative effectiveness (beta=.59, *P*<.001): the intervention had more positive effects on relative effectiveness for those with high education compared with those low in education. There was no significant interaction between condition and sample on any of the outcome measures (*P* ≥.04). For the interaction effects between intention at baseline and the outcome measures, See Table 4. If an OR is higher than one, this means that the higher the score on a factor, the higher the outcome of IDM or the higher the chance of the daughter being vaccinated. If an OR is less than one, this means that the higher the score on a factor, the lower outcome of IDM or the lower the chance of the daughter being vaccinated. In the first 2 columns, the reference category is those with a negative intention. For a comparison between those in doubt (reference category) and a positive attitude, see the third column. Significant interaction effects between intention at baseline and condition were found on intention, attitude, decisional conflict, subjective norm, and relative effectiveness . For mothers who had a negative intention, the intervention had more positive effects on intention and relative effectiveness compared with mothers who were doubting (beta=.26, *P*=.002; beta=.39, *P*=.001, respectively) or had a positive intention (beta=.40, *P*<.001; beta=.53, *P*<.001, respectively). In addition, for mothers with a negative intention, the intervention had a more positive effect on attitude (beta=.21, *P*=.001) and on subjective norms (beta=1.64, *P*<.001) compared with mothers with a positive intention. For mothers who were doubting, the intervention had more positive effects on decisional conflict compared with mothers who had a negative intention (beta=.26, *P*=.001). No differences on intervention outcomes were found between mothers who were doubting and mothers who had a positive intention (*P* ≥.004).

**Table 4:** Moderation effects of intention subgroups on the outcome measures according to the intention-to-treat analyses (N=8062).

Outcome			Negative—in doubt			Negative—positive			In doubt—positive	
			Beta (standard error)	*P* value	Beta (standard error)		*P* value	Beta (standard error)		*P* value
Primary outcome										
	**HPV** ^a^ **vaccination uptake**									
		Has received no HPV injection (reference)								
		Has received one or two HPV injections	−.21 (.13)	.22	.01 (.18)		.97	.22 (.18)		.24
Secondary outcomes										
	**IDM** ^b^ **: dichotomous**									
		Not informed (reference)								
		Informed	.18 (.15)	.22	.09 (.15)		.56	−.10 (.14)		.50
	IDM: continuous (0-48)		.92 (.69)	.18	1.03 (.65)		.12	.11 (.65)		.87
	Decisional conflict (1-7)		.26 (.08)	.001^c^	.04 (.07)		.57	.22 (.08)		.004
	Intention (1-7)		−.26 (.08)	.002^c^	−.40 (.08)		<.001^c^	−.14 (.07)		.03
	Attitude (1-7)		−.17 (.06)	.009	−.21 (.06)		.001^c^	−.04 (.06)		.51
	Beliefs (1-7)		−.04 (.04)	.36	−.02 (.04)		.68	.02 (.04)		.67
	Risk perception not vaccinated (1-7)		.04 (.07)	.51	.09 (.07)		.18	.05 (.06)		.41
	Risk perception vaccinated (1-7)		.04 (.06)	.51	−.06 (.07)		.38	−.10 (.07)		.17
	Anticipated regret (1-5)		−.03 (.07)	.70	.02 (.07)		.81	.04 (.06)		.46
	Subjective norm (−10 to 10)		−1.18 (.47)	.01	−1.64 (.43)		<.001^c^	−.46 (.37)		.22
	Habit (1-7)		.08 (.08)	.34	.06 (.08)		.45	−.01 (.07)		.87
	Relative effectiveness (1-10)		−.39 (.12)	.001^c^	−.53 (.12)		<.001^c^	−.14 (.11)		.21
	Self-efficacy (1-7)		−.00 (.05)	.97	.03 (.04)		.45	.03 (.05)		.50
	Knowledge (−8 to 8)		−.01 (.13)	.92	−.13 (.12)		.29	−.11 (.12)		.34

^a^HPV: human papillomavirus.

^b^IDM: informed decision making.

^c^*P*<.003, thus significant (Bonferroni: 0.05/14 factors).

### Subjective Program Evaluation and Objective Program Use

Mothers in the intervention condition evaluated the website with a 7.6 (SD=1.36) and the virtual assistants with a 7.4 (SD=1.53). According to the computer logs, 2509 (62.80%) of the 3995 (100.00%) invited mothers logged on to the website. Of these, 1835 (73.14%) visited the website once, 498 (19.84%) visited twice, and 176 (7.02%) more than twice. On average, mothers spent 22 min on the website (SD=13 min).

## Discussion

### Principal Findings

This study investigated the effectiveness of a Web-based tailored intervention with virtual assistants promoting HPV vaccination acceptability among mothers of invited girls. As hypothesized, positive intervention effects were found with respect to the social cognitive determinants of the mothers’ decision making about the vaccination (eg, HPV vaccination-related intention, attitude, and outcome beliefs), levels of IDM, and levels of decisional conflict. The positive effect of tailored education on HPV vaccination intention was also found by Gerend et al [12] among young women. However, they did not assess other determinants of HPV vaccination acceptability (next to intention) nor did they measure levels of IDM, levels of decisional conflict, or actual HPV vaccination uptake.

The findings described above suggest that this intervention has potential in promoting HPV vaccination acceptability and IDM. This is important, given the currently moderate HPV vaccination uptake and the fact that large proportions of the mothers do not actively acquire and process information about the pros and cons of this HPV vaccination and that many feel ambivalent about the decision [7,8]. Less informed decisions are decisions constituted in rather instable beliefs that are susceptible to counterarguments. Nowadays, counterarguments are all around on the Internet and Web-based social media [48]. Because the intervention initiated active processing of verifiable information about the risks and effectiveness of the HPV vaccination, it inoculates mothers with arguments that become accessible at the moment they are confronted with (new) information that might challenge their initial positive attitudes and intentions [49,50].

No effects were found on mothers’ perceived risk of their daughter getting cervical cancer without the HPV vaccination, anticipated regret in case their daughter would get cervical cancer later in life, and self-efficacy. As for risk perception, baseline scores indicated that the mothers overestimated the probability of contracting cervical cancer to a great extent when taken into account the actual population incidence [51]. Because the intervention presented mothers this actual low probability of attracting cervical cancer, it seems unlikely that their perceived risk was brought to higher levels. The lack of effect on anticipated regret might be explained by the fact that we removed the intervention component specifically targeting anticipated regret. This was removed because our pilot studies and focus groups revealed that resistance was evoked by asking mothers how much regret they would have if they did not vaccinate their daughter against HPV and their daughter developed cervical cancer later in life. Also, emphasizing the impact of cervical cancer might be fear-arousing, which, in turn, may have been detrimental for exploring and processing other information provided by the program [52]. Finally, it appeared that we encountered a ceiling effect for self-efficacy as the scores at baseline among both groups were above 6 on a 7-point scale.

No effects of the tailored intervention were found on HPV vaccination uptake. This is contrary to both our expectations and to what has been found by others; Hopfer found that among female college students, HPV vaccination uptake doubled after they were exposed to a tailored video compared with controls (22% vs 12%, respectively) [11]. However, in their study, vaccination rates were quite low in the control condition (12%), leaving much room for improvement. In our study, however, the uptake rates were high in both conditions (intervention: n=3995; 73.17% and control: n=4067; 72.71%) especially when compared with the national Dutch uptake (n=59,866; 60.98) [6]. This may explain why we, as opposed to Hopfer, did not find an effect on HPV vaccination uptake. After all, we did find a larger increase in intention among mothers in the intervention compared with the control condition, and according to theory [23] and empirical findings [53], intention is an important predictor of (HPV vaccination) behavior.

Mothers evaluated the intervention as positive, specified by the high subjective evaluation of both the website (7.6 on a 10-point scale) and the virtual assistants that were used to deliver the tailored feedback (7.4 on a 10-point scale). Objective program use was also high, with 62.80% (n=2509) of the invited mothers having visited the website. In addition, on average, they spent quite some time on the website (22 min). Taken together, the intervention has potential for broad national dissemination and implementation.

Furthermore, subgroup analysis with 3 intention groups (ie, negative, in doubt, and positive) showed that the intervention had the most positive effects on decisional conflict for mothers who were doubting. For mothers with a negative intention, the most positive effects were found on intention, attitude, and subjective norms. This is promising, as for a population-wide program it is relevant to guide those in doubt toward making an informed choice without decisional conflict and to persuade those having negative intentions toward vaccination. Fortunately, we did not find any adverse effects of the intervention in mothers with a negative intention, such as a further decrease in their intention or attitude.

### Methodological Considerations

There are two methodological considerations. First, with large sample sizes, as in this study, even small effects can become statistically significant [54]. However, the positive intervention effects were consistently found on almost all outcomes. In addition, the large sample provided us with sufficient data for conducting analyses on subsamples (ie, based on sociodemographics, sample, and three intention groups) while maintaining sufficient levels of power [54]. The effect sizes that we found are in line with other Web-based interventions targeting health behavior outcomes [55]. We believe these, even small effects, are of relevance in public health as they become substantial at the population level. In addition, the intervention was of help for those in doubt and did not have any detrimental effect. We therefore find this Web-based tailored program is a substantial step forward in improving both research and practice in the context of the promotion of HPV vaccination acceptability.

Second, we used a scale comprising two items of the Self-Report Habit Index for measuring habit [41]. This accounted for the extent to which getting the HPV vaccination was something mothers did (1) naturally and (2) without thinking. The positive intervention effect on the composite measure might indicate that the intervention induced perceptions about the HPV vaccination as something you take for granted, without thinking. The latter is unwanted, considering the aim of initiating active processing of information about the vaccination. Fortunately, secondary analysis separating the two items showed that there was a positive effect of the intervention on the first item (ie, “naturally”) but not on the second (ie, “without thinking”). In retrospect, the label “habit” attributed to the two-item operationalization appears misleading, though the intervention strengthened the mothers’ belief about getting the HPV vaccination as something natural.

### Strengths and Limitations

Important strengths of this study are the randomized controlled design, adequate sample sizes, and the reliable objective assessment of HPV vaccination uptake. Furthermore, the systematic, stepwise development of the intervention was a notable strength, as well as the mothers’ positive subjective evaluation of the intervention and the objectively assessed high level of program use.

Some limitations of this study should be considered as well. First, the study was subject to a considerable amount of attrition. Unfortunately, attrition is quite common in studies on Web-based interventions [56]. Dropout analysis showed that dropout was selective. For instance, there were higher dropout rates in the intervention condition, which has also been reported for other (tailored) interventions [57-59]. In this study, we handled the missing data and selective dropout by using multiple imputation [60]. Results from the complete case analyses only slightly differed from those from the ITT analyses. Thus, it seems unlikely that the observed effects are spurious or due to selective dropout. Second, caution is needed when generalizing the results of this study to the general population (ie, Dutch mothers of girls aged 12 years) because we were unable to check the sample’s representativeness. However, we did not find any differences in effectiveness of the intervention in specific subgroups of participants, as indicated by the conducted moderation analyses with sociodemographics.

### Conclusions and Recommendations

The study findings suggest that this Web-based tailored intervention has the potential to improve both HPV vaccination acceptability and IDM, and decrease decisional conflict among mothers of invited girls. Therefore, we recommend nationwide dissemination and implementation. Furthermore, we recommend developing (tailored) interventions targeting the daughter and the mother’s partner, as these have appeared to be important social referents [7,8]. Research has indicated that tailoring the intervention could have beneficial effects for girls, as they also expressed their need for interactive and personal information about the HPV vaccination [8]. This still needs to be investigated for the partners. In addition, boys may also become a relevant target group. In other countries, such as Australia, boys are already included in the national immunization program. This may contribute to the achievement of herd immunity and to a reduction of the global burden of a variety of HPV-related cancers in women and men [61-63].

## Appendix

Multimedia Appendix 1 Intervention screenshots.

Multimedia Appendix 2 CONSORT-EHealth (V 1.6.1).

## Abbreviations

HPV: human papillomavirus
IDM: informed decision making
ITT: intention-to-treat analysis
NIP: National Immunization Program
RCT: randomized controlled trial
SD: standard deviation
