# Correction: Effectiveness of a Web-Based Tailored Intervention With Virtual Assistants Promoting the Acceptability of HPV Vaccination Among Mothers of Invited Girls: Randomized Controlled Trial

**DOI:** 10.2196/22565

**Published:** 2020-07-28

**Authors:** Mirjam Pot, Theo GWM Paulussen, Robert AC Ruiter, Iris Eekhout, Hester E de Melker, Maxine EA Spoelstra, Hilde M van Keulen

**Affiliations:** 1 Netherlands Organization for Applied Scientific Research (TNO) Child Health Leiden Netherlands; 2 Department of Work and Social Psychology Maastricht University Maastricht Netherlands; 3 VU University Medical Center Epidemiology & Biostatistics Amsterdam Netherlands; 4 National Institute for Public Health and the Environment (RIVM) Centre for Infectious Disease Control Bilthoven Netherlands; 5 Department of Psychology Leiden University Leiden Netherlands

In “Effectiveness of a Web-Based Tailored Intervention With Virtual Assistants Promoting the Acceptability of HPV Vaccination Among Mothers of Invited Girls: Randomized Controlled Trial” J Med Internet Res 2017;19(9):e312 the authors noted several errors.

In the Methods section, under the subheading “Statistical Analyses, the phrase:

Effect sizes of the linear regressions were calculated in R (R Development Core Team) [45] using Cohen ]ƒ^2^ (*R*^2^ including the outcome at baseline and condition/1− *R*^2^ only including the outcome at baseline).

Has been revised to:

Effect sizes for the linear regressions were calculated in R (R Development Core Team) [45] using Cohen ƒ^2^ statistic, (*R*^2^*_AB_* – *R*^2^*_A_*)/(1 – *R*^2^*_AB_*), in which *B* is the variable of interest (ie, condition), *A* is the set of all other variables (ie, the outcome at baseline), *R*^2^*_AB_* is the proportion of variance accounting for *A* and *B* together, and *R*^2^*_A_* is the proportion of variance accounted for by *A*.

This change has not affected the results of the paper.

Additionally, the following degrees have been revised: Mirjam Pot’s degree has been changed from MSc to PhD, Maxine ME Spoelstra’s degree has been changed from BSc to MSc, and Robert AC Ruiter’s degree has been changed from PhD to Prof Dr.

Finally the Corresponding Author section has been revised. Mirjam Pot's email address has been updated from mirjam.pot@tno.nl to mirjampot90@gmail.com. The phone number has been updated from "31 888662213" to "31 643234293", and the fax number (31 888660613) has been removed.

The correction will appear in the online version of the paper on the JMIR Publications website on July 28, 2020, together with the publication of this correction notice. Because this was made after submission to PubMed, PubMed Central, and other full-text repositories, the corrected article has also been resubmitted to those repositories.

