# Supplementary material for: Effectiveness of a Web-Based Tailored Intervention With Virtual Assistants Promoting the Acceptability of HPV Vaccination Among Mothers of Invited Girls: Randomized Controlled Trial
Source: J Med Internet Res. 2017 Sep 6;19(9):e312. doi: 10.2196/jmir.7449 (PMC5607435; doi:10.2196/jmir.7449)

Multimedia Appendix 1

Screenshots of the website

Picture 1: The first menu of the website ('information about the HPV-vaccination').

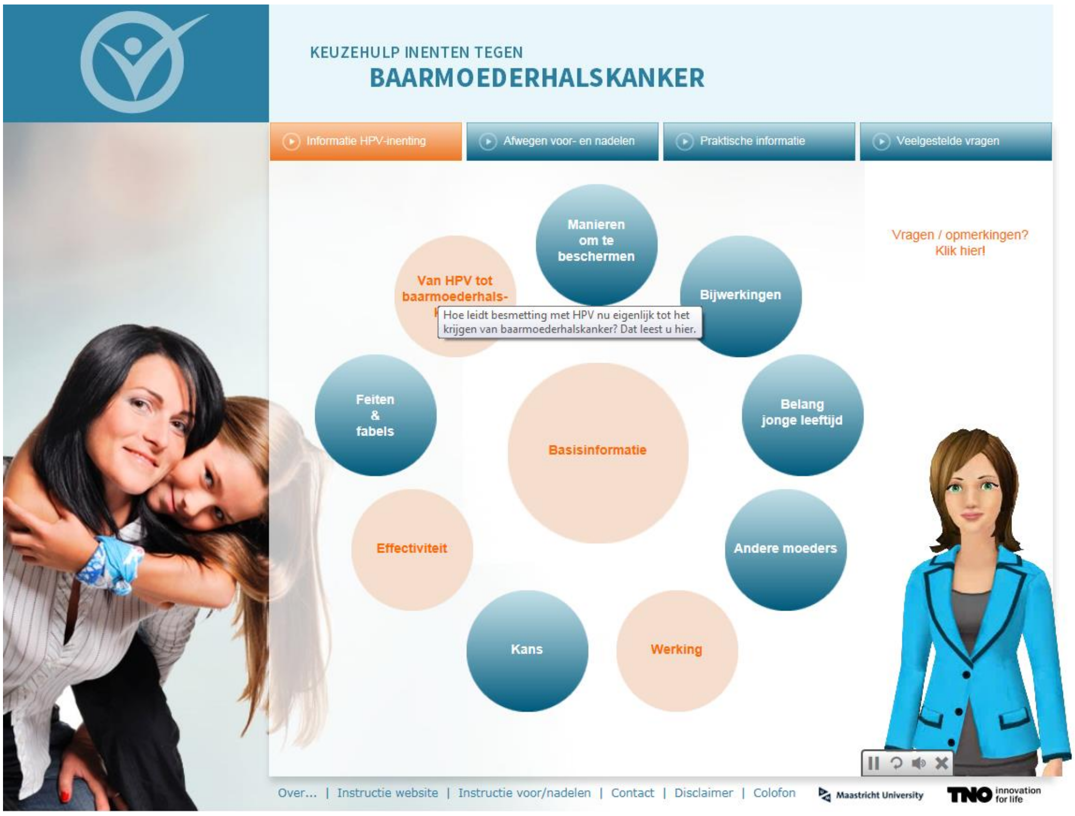

Picture 2: Within the 'risk component' of menu 1, the assistant asks a question about a mothers' risk perception of her daughter getting infected with HPV on which feedback was then tailored (Picture 3).

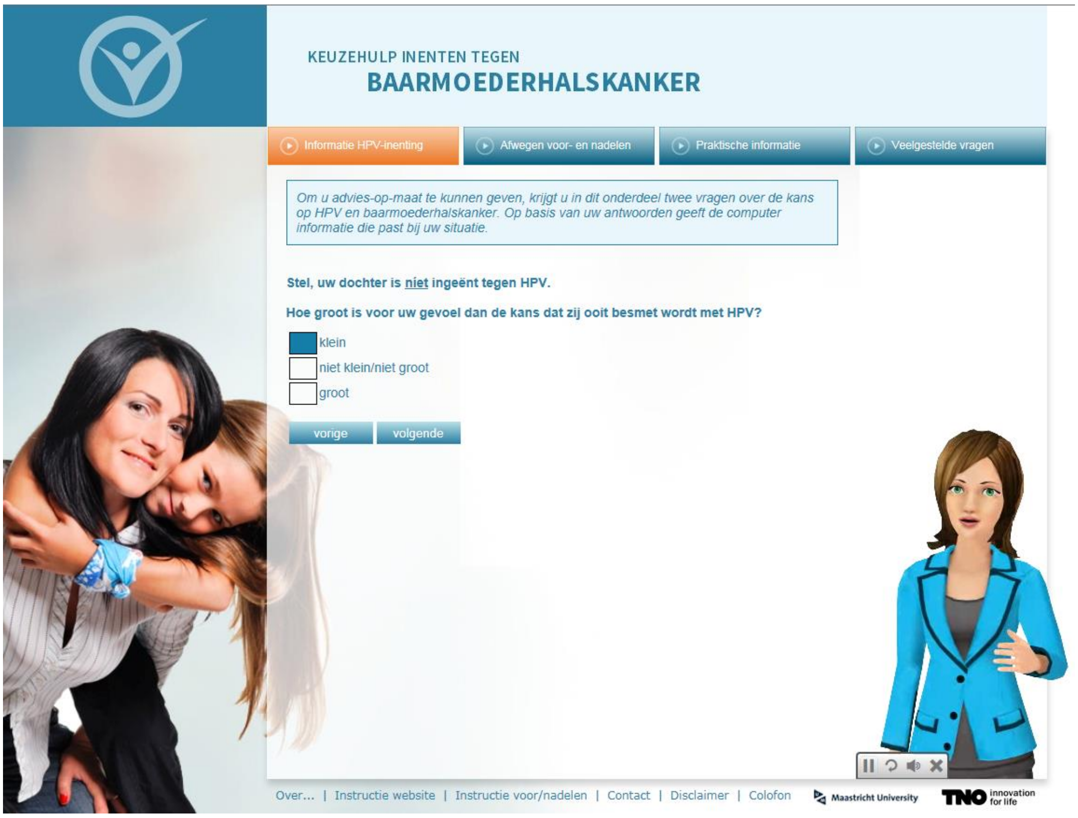

Picture 3: Tailored feedback on mothers' risk perception by the doctor-like assistant.

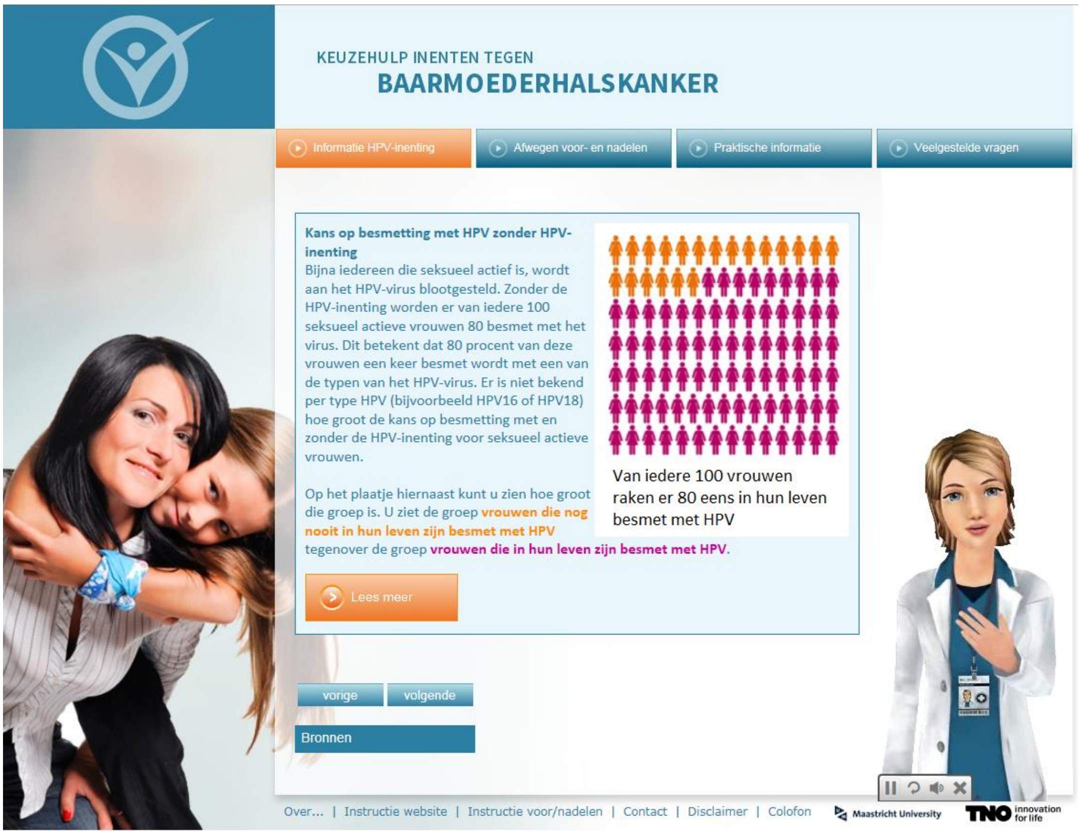

Picture 4: The third menu of the website ('practical information').

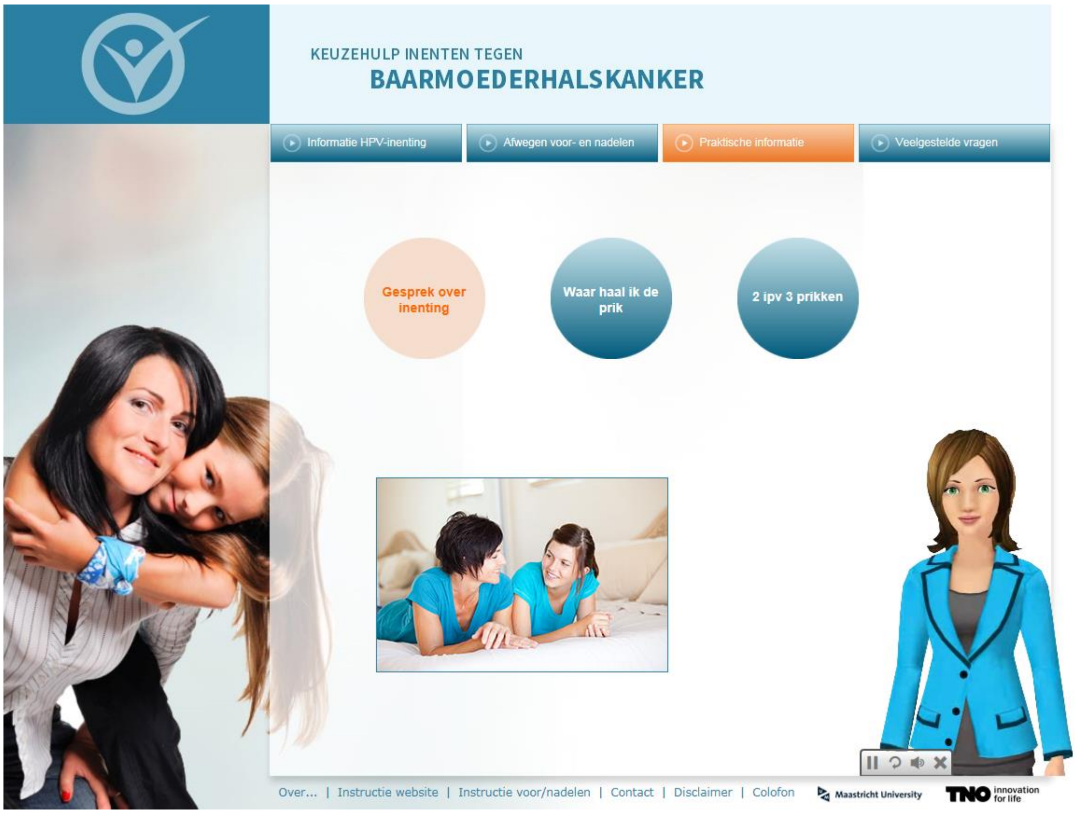

Picture 4: The fourth menu of the website ('frequently asked questions').

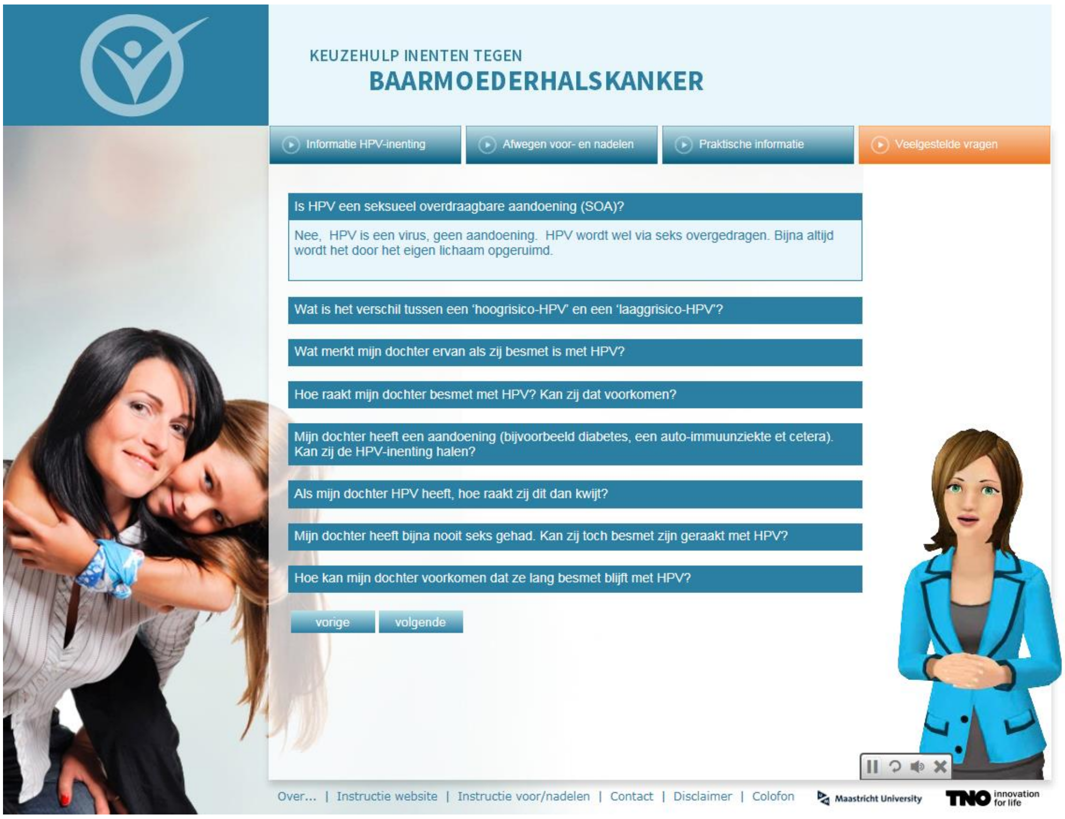

Supplement: Multimedia Appendix 1 [file jmir_v19i9e312_app1.pdf]
